# Comprehensive evaluation of the allelopathic potential of *Elymus nutans*

**DOI:** 10.1002/ece3.7982

**Published:** 2021-08-24

**Authors:** Xiaolong Quan, Youming Qiao, Mengci Chen, Zhonghua Duan, Huilan Shi

**Affiliations:** ^1^ State Key Laboratory of Plateau Ecology and Agriculture Qinghai University Xining China; ^2^ College of Ecol‐Environmental Engineering Qinghai University Xining China

**Keywords:** allelopathy, alpine meadow, autotoxicity, gramineae, grassland degradation, mixed sowing

## Abstract

*Elymus nutans* has been widely planted together with other perennial grasses for rebuilding degraded alpine meadow atop the Qinghai‐Tibetan Plateau. However, the rebuilt sown pastures begin to decline a few years after establishing. One of the possible causes for the degradation of sown grassland may come from allelopathy of planted grasses. The purpose of this study was to examine allelopathic potential of *Elymus nutans*. Three types of aqueous extract from *Elymus nutans* and its root zone soil were prepared, and 5 highland crops and 5 perennial grasses were used as recipient plants. *Elymus nutans* exhibited strong allelopathic potential on germination and seedling growth of 5 crops, but different crops or perennial grasses respond to the extract differently. The pieces aqueous extract have stronger inhibition than whole plant extract and root zone soil extract. *Hordeum vulgar* var. *nudum*, *Avena sativa,* and *Festuca sinensis* were the most affected, while *Chenopodium quinoa* and *Elymus sibiricus* were the least affected. *Elymus nutans* presented less influence on *Poa pratensis* and *Poa crymophylla* than on *Festuca sinensis*. It is recommended that the species combination of mixture for restoration should be considered for allopathic effects on the coseeding to decrease the seeding rate ratio of *Elymus nutans*. The annual dicot crop seeds of *Chenopodium quinoa* and *Brassica napus* can be used as alternative subsequent crop for the seed field of *Elymus nutans* monoculture.

## Graphical abstract

Seeds, including 8 species of monocotyledon and 2 species of dicotyledon, were tested. (Photograph by XLQ)

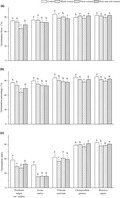

## 1 INTRODUCTION

Ecological projects are essential for the adaptation and restoration of ecosystems in response to environmental changes and human disturbances (Cai et al., 2015; Dong et al., 2020). Revegetation not only contributes to the naturalization of the landscape but also reduces soil erosion and increases carbon capture and recreational functions of the territory (Lasanta et al., 2015). Degradation of alpine meadow atop Qinghai‐Tibetan Plateau (QTP) has been one of great concerns of academy community, pastoralists, and government officials over past four decades (Dong et al., 2020; Qin, 2014). One of the characteristics of degraded alpine meadow is that the sedge‐dominant vegetation is replaced by unpalatable or poisonous forb‐dominated vegetation (Santonja et al., 2019). The severely degraded alpine meadows are characterized by fragmented turf with denudated patches. Many trials have been conducted to select suitable forages that can be used in the alpine meadow region but turned out that only some gramineous perennial grass can adapt to the local natural condition (Zhang et al., 2015, 2017). No suitable perennial legumes have been selected to apply to the ecological restoration of degraded meadow (Shang et al., 2017).

Due to the impossibility to restore degraded alpine meadows with native dominant *Kobresia* plants, mix‐seeding(mixture) gramineous perennial grass has been regarded as an ideal option for the revegetation of degraded alpine meadow on the QTP because it increases the diversity and stability of the planted community (Ma et al., 2002; Shi et al., 2009), though the pristine alpine meadow vegetation communities are mainly composed of plant species of *Kobresia* family, such as *Koresia pygmaea and Koresia humilis* (Qiao & Duan, 2016). Planting perennial grasslands on degraded alpine meadow can not only increase the land utilization rate and restore the degraded grassland vegetation as soon as possible but also ease the grazing pressure on natural grasslands and prevent grassland degradation and desertification.

The primary grasses being planted for revegetating severely degraded alpine meadow are limited to few graminoid varieties, such as *Elymus nutans*, *Poa crymophila*, *Poa pratensis,* and *Festuca sinensis*. Among these germplasms, *Elymus nutans* is the major seed material for ecological restoration, and the ratio of seeding rate of *Elymus nutans* in mixture accounts for 50%. This reflects *Elymus nutans*' relative ease of establishment, high forage production, and sufficient seed supply.

However, the established grassland with perennial grasses begins to decline within 2–4 years, causing economic and ecological loss (Dong et al., 2007). Some sown grasslands are degraded severely (bare land), challenging the sustainable use of revegetated grassland in alpine regions (Shang et al., 2006). It is recorded that most monocropped perennial grasses can be utilized for around ten years in low‐altitude areas, but the yield of monoculture of perennial grasses in plateau area begins to decline 3 years after planting which is shorter than expected (Dong et al., 2010).

It is undeniable that redegradation of sown pasture on the plateau may be caused by a combination of biotic and abiotic stresses. The interspecies (intraspecies) competition may be an important cause (Lin et al., 2018; Uddin et al., 2020). The authors argue that the decline or redegradation of vegetated grassland resulted from interspecies competition between mix‐seeding (coseeding) plants and between grasses and unpalatable forbs. The degradation of *Elymus nutans* monoculture may result from autotoxicity.

Given the fact that much research into plant allelopathy focused on farmland crops and turfgrasses (Yu et al., 2020), and little information about allelopathy of perennial gramineous forage grass is available, we focus our attention on allelopathic effects of *Elymus nutans* on seed germination and seedling growth of mix‐seeding plants and itself. We suppose that the concentration of allelopathicals is below the threshold value of inhibiting in the early stage of the establishment of community or monoculture and is conducive to plants that are invaded or coplanted. With the growth year, the allelopathicals accumulate in the soils, and *Elymus nutans* not only outcompete coseeding grasses but also release allelopathicals, which enter the soil by leaching and inhibit the germination and the growth of seedlings of itself. The competitiveness of *Elymus nutans* is weakened, which provides opportunities for invasion of unplatable plants and further accelerates the degradation of seeded pasture and replacement of unwanted vegetation.

The objectives of this study were (a) to evaluate the allelopathical potential of *Elymus* on highland crops, coseeding grasses, and *Elymus* itself; and (b) to reveal the potential cause of sown grassland degradation and provide reference for sustainable use and management of revegetated grassland in alpine region.

## 2 MATERIALS AND METHODS

### 2.1 Plant and soil materials

Grass seeds of *Elymus nutans*, *Elymus sibiricus*, *Festuca sinensis*, *Poa pratensis,* and *Poa crymophila* were harvested in September 2019 and were provided by Tongde Forage Seed Production Base (35°15′N, 100°38′E) of Qinghai Province, which is located at the eastern QTP. Crop seeds of *Hordeum vulgar* var. *nudum*, *Avena sativa*, *Triticum aestivum*, *Chenopodium quinoa,* and *Brassica napus,* which were harvested in 2019, were acquired from the Academy of Agriculture and Forestry Sciences, Qinghai University. These crops can grow well in the agricultural area of the province. All seeds were stored in the refrigerator at 4℃ before experiment. Plants of *Elymus nutans* were collected in early August 2019, when the plant growth was in peak period. Robust whole *Elymus nutans* plants with roots were dug out with a small shovel. The plants were cut into aerial parts and belowground parts on site. The root zone soils were collected by shaking the roots parts. All plant and soil materials were put in plastic bags and kept in a refrigerator and taken back to the laboratory for experiment.

### 2.2 Preparing of extract

Two kinds of plant extract were prepared. The whole plant extract was prepared by soaking whole fresh aboveground parts in distilled water (w/v, 1:4). The pieces aqueous extract were prepared by cutting plants into 1–2 cm and soaking them in distilled water (w/v, 1:4). The containers of soaked plant materials were shaken at 500 rpm for 5 min per 12 hr in 72 hr at room temperature. Soil extract was prepared by soaking fresh root zone soil in distilled water (w/v, 1:2) and shaken at 200 rpm for 24 hr in gyratory shaker at room temperature.

The resulting mixtures were passed through qualitative filter paper and 0.45‐μm aqueous membrane, respectively. Thus, 0.25 g/ml plant extract and 0.5 g/ml root zone soil extract were obtained. The solutions were stored at 4℃ until use.

### 2.3 Germination experiment

All seeds were surface‐sterilized by soaking in 0.5% sodium hypochlorite solution for 10 min and rinsed several times with distilled water. Five replicates of 50 seeds were placed in Petri dishes (90 × 15 mm) lined with 2‐layer filter paper. The seeds and filter paper were wetted with 3 ml distilled water or extract solution. To maintain moisture, the petri dishes were put in plastic bags and placed in a growth chamber at temperature of 20/16℃ for 12:12 h (light/dark) period. The light intensity was 3,000 lux. A seed was considered germinated when the root protruded ≥2 mm. Germination was counted at 24‐hr interval over 15 days, and the first count was carried out on the fourth day.

### 2.4 Seedling growth experiment

To evaluate the effects of different extract on seedling growth, a completely randomized block design with three replications was applied to conduct seedling growth tests on growth plate (5 × 8 cups; Ø 40 mm; height 80 mm). The media were a mixture of vermiculite/pearlite (50:50, v/v). Three pregerminated seeds (5 days old) were transplanted into each cup, and ten cups were used as a replicate. The growth plates were placed in growth chambers with temperature of 20/16℃ for 12:12 h (light/dark) period. The light intensity was 3,000 lux. The moisture content of the media was maintained at a ratio of 1.5:1 solution or water to media on weight basis by spraying different plant extract every three days. After 10 days of growth, the seedling with roots was carefully washed from media. Ten seedlings of a replicate were selected, and the plumule length and radical length of each seedling were measured. The seedlings were oven‐dried to constant weight at 65℃ and weighed to obtain dry weights.

### 2.5 Data analysis

Germination force (GF), germination percentage (GP), and germination index (GI) were calculated according to the following equation (Wang et al., 2019):

$$\text{GF}\;\left(\%\right)=\text{Number of germinated seeds in first one - third of evaluation days}/\text{Total number of Seeds}\times 100$$

$$\text{GP}\;\left(\%\right)=\left(N_{1}+N_{2}+N_{3}\ldots +N_{n‐1}+N_{n}\right)/\text{Total number of Seeds}\times 100$$

$$\text{GI}=\left(N_{1}\right)/1+\left(N_{2}\right)/2+\left(N_{3}\right)/3+\ldots +\left(N_{n‐1}\right)/\left(n‐1\right)+\left(N_{n}\right)/n$$

where *N*_1_, *N*_2_, *N*_3_,…, *N_n_*: the number of germinated seeds in the first, second, and final counts, and 1, 2,…, *n* was the first, second, and final evaluation days.

The allelopathic effect response index (RI) and comprehensive allelopathic effect response index (CE) were calculated using the equation suggested by Williamson and Richardson (1988):

RI=1‐C/T(T≥C)orC/T‐1(T<C)

$$\text{CE}=({\text{RI}}_{1}+{\text{RI}}_{2}+{\text{RI}}_{3}+\ldots +{\text{RI}}_{6})/6$$

where *T* is the treatment value, *C* is the corresponding value of control, and RI_1_ to RI_6_ were allelopathic indexes of GF, GP, GI, root length (RL), shoot length (SL), and dry weight (DW), respectively. Positive values indicate stimulating effects, while negative ones indicate inhibitory activity of the aqueous extract.

Two‐way analysis of variance (ANOVA) model was used to test plant species, extract effects, and the species ×extract interaction effects. One‐way ANOVA was used to compare treatment effects of different extract on the same recipient plant. Statistically significant difference was assumed at the probability level of 0.05, and means were separated by using LSD.

## 3 RESULTS

### 3.1 Allelopathic potential of *Elymus nutans* on highland crops

Seed germination (GF, GP, and GI) and seedling growth (SL, DW) of the highland crops were significantly affected by crop species (A), aqueous extract type (B) of *Elymus nutans*, and their interactions (A × B). RL of the highland crops was significantly influenced by crop species and aqueous extract type but not by their interaction (Table 1).

**TABLE 1:** Analysis of variance for examining highland crop, extract, and crop × extract interaction effects

	*df*	GF		GP		GI	
		*F*	*p*	*F*	*p*	*F*	*p*
Highland crop	4	53.057	.000**	121.420	.000**	228.395	.000**
Extract	3	8.793	.000**	45.277	.000**	10.392	.000**
Crop × extract	12	4.252	.000**	4.359	.000**	2.201	.019*

	*df*	RL		SL		DW	
		*F*	*p*	*F*	*p*	*F*	*p*
Highland crop	4	956.645	.000**	2,597.983	.000**	13,295.077	.000**
Extract	3	18.559	.000**	34.927	.000**	92.442	.000**
Crop × extract	12	1.713	.079 ns	4.592	.000**	13.649	.000**

Abbreviations: A, species; B, aqueous extract of *Elymus nutans*; *df*, degrees of freedom; ns, not significant.

*Significant at *p* < .05.

**Significant at *p* < .01.

**FIGURE 1:**
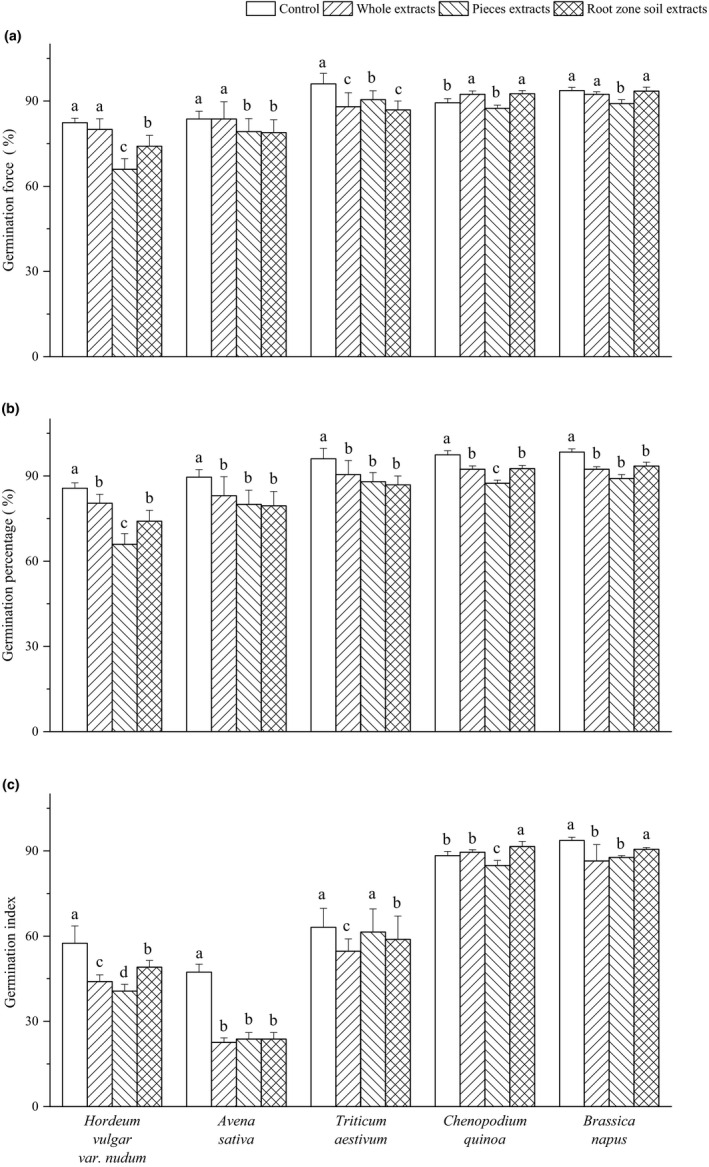
Effect of *Elymus nutans* extract on germination of highland crops. Values are mean ± *SD* (*n* = 5). Different letters denote significant differences between treatments with a > b > c > d

GF of the tested crop seeds to different extract was dependent on crop varieties and extract types (Figure 1a). Compared with control, the whole extract of *Elymus nutans* had no significant effect on the GF of *Hordeum vulgar* var. *nudum*, *Avena sativa,* and *Brassica napus*, but decreased GF of *Triticum aestivum* and increased that of *Chenopodium quinoa*. Significant inhibitory effects of pieces aqueous extract on the GF of *Hordeum vulgar* var. *nudum*, *Avena sativa*, *Triticum aestivum*, and *Brassica napus* were detected except for *Chenopodium quinoa*. Root zone soil extract reduced the GF of *Hordeum vulgar* var. *nudum*, *Avena sativa*, *Triticum aestivum,* and *Brassica napus* and increased that of *Chenopodium quinoa*.

All the three kinds of extract significantly reduced the GP of tested crops as shown in Figure 1b. *Avena sativa*, *Triticum aestivum,* and *Brassica napus* presented similar pattern, and their GP decreased by three kinds of extract compared with control, but there were no differences among the extract treatments. The pieces aqueous extract of *Elymus nutans* significantly inhibited the germination of *Hordeum vulgar* var. *nudum* and *Chenopodium quinoa*. *Hordeum vulgar* var. *nudum* germination was sensitive to pieces aqueous extract.

Comparably, the three types of extract have lesser effect on the GI of *Brassica napus* and *Chenopodium quinoa*, but they had greater impact on that of *Hordeum vulgar var. nudum*, *Avena sativa,* and *Triticum aestivum* (Figure 1c). The root zone soil extract could improve the GI of *Chenopodium quinoa*, but no effects on *Brassica napus*. Both whole plant extract and pieces aqueous extract could significantly decrease the GI of *Hordeum vulgar* var. *nudum* and *Avena sativa*. The effects of the three types of extract on the GI of *Triticum aestivum* varied.

**FIGURE 2:**
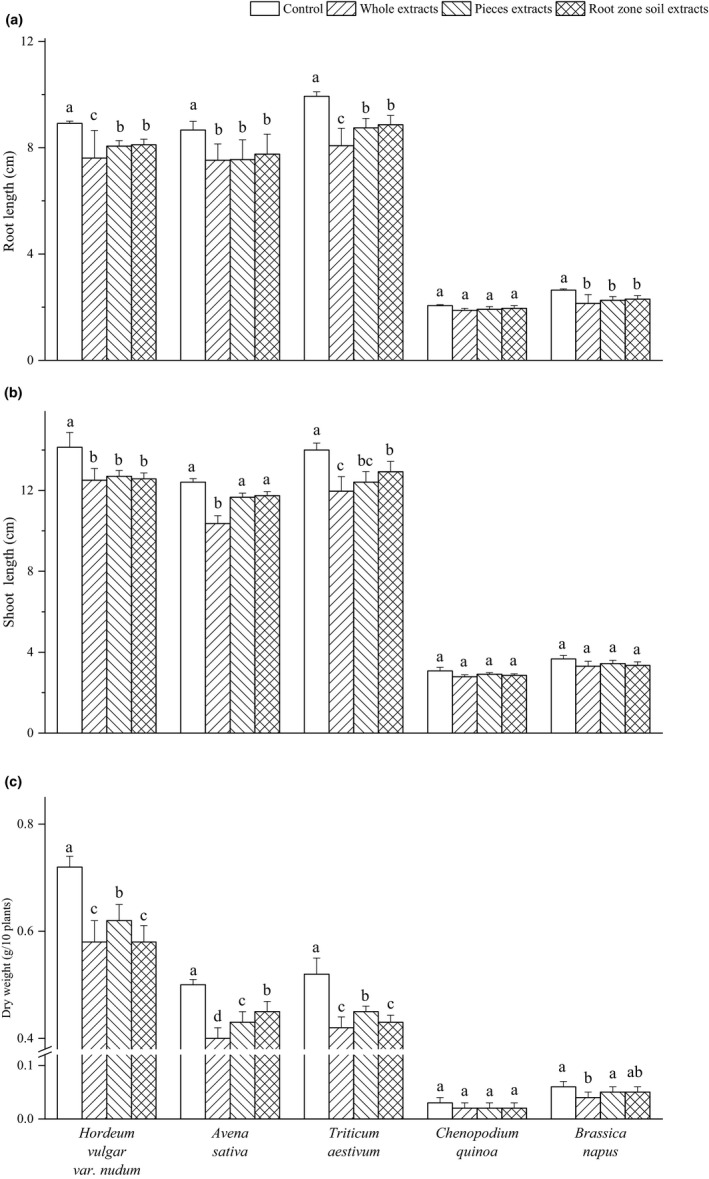
Effects of *Elymus nutans* extract on growth of highland crops. Values are mean ± *SD* (*n* = 5). Different letters denote significant differences between treatments with a > b > c > d

RL of *Hordeum vulgar* var. *nudum*, *Avena sativa*, *Triticum aestivum,* and *Brassica napus* showed significant differences under three kinds of extract, while there was no significant effect on *Chenopodium quinoa* (Figure 2a). The whole extract exhibited stronger inhibitory effects than pieces and root zone soil extract on root growth of *Hordeum vulgar* var. *nudum* and *Triticum aestivum*. No significant difference in root growth was detected among the three extract treatments for *Avena sativa* and *Brassica napus*.

The SL of *Hordeum vulgar* var. *nudum*, *Avena sativa,* and *Triticum aestivum* was impeded by the three kinds of extract, whereas *Chenopodium quinoa* and *Brassica napus* shoot were not sensitive to the extract (Figure 2b). There was no significant difference in the SL of *Hordeum vulgar* var. *nudum* among whole, pieces, and root zone soil extract. *Avena sativa* shoot proved to be sensitive to the whole extract of donor plants. The extract of whole plant, pieces, and root zone soil exhibited 14.55%, 11.32%, and 7.71% of allelopathic inhibition on the SL of *Triticum aestivum*.

Similar to the effects of extract on SL and RL, seedling DW of *Hordeum vulgar* var. *nudum*, *Avena sativa*, *Triticum aestivum,* and *Brassica napus* to three kinds of extract showed differences with crops and extract (Figure 2c). No significant difference was detected in dry weight of *Chenopodium quinoa* among different extract treatments. The whole and root zone soil extract exhibited higher inhibition than the pieces aqueous extract on seedling DW of *Hordeum vulgar* var. *nudum* and *Triticum aestivum*.

**FIGURE 3:**
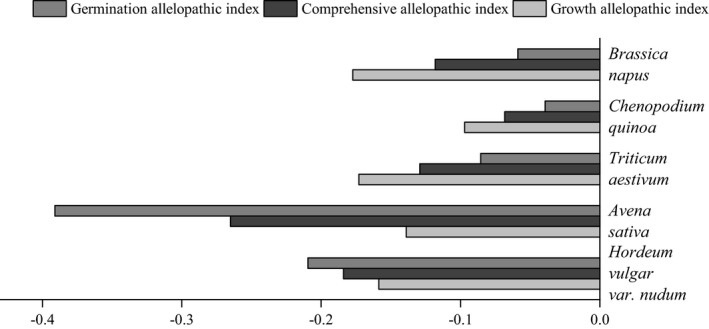
Comprehensive allelopathic effect index of *Elymus nutans* extract on highland crops

The allelopathic effect response index and comprehensive allelopathic effect response index of *Elymus nutans* on the tested crops are presented in Figure 3. The inhibition of the extract on *Hordeum vulgar* var. *nudum* and *Avena sativa* occurred in germination with germination allelopathic index of −0.391 and −0.210, respectively. *Triticum aestivum*, *Chenopodium quinoa,* and *Brassica napus* occurred in seedling growth with growth allelopathic index of −0.173, 0.097, and −0.177, respectively. *Avena sativa* exhibited the largest comprehensive allelopathic index of −0.265, whereas *Chenopodium quinoa* presented the smallest one (−0.068).

### 3.2 Autotoxicity of *Elymus nutans* and its allelopathic potential on perennial grasses

Seed germination and seedling growth of the perennial grasses were significantly affected by species (A), aqueous extract type (B) of *Elymus nutans,* and their interactions (A × B, Table 2).

**TABLE 2:** Analysis of variance for examining grass, extract, and grass × extract interaction effects

	*df*	GF		GP		GI	
		*F*	*p*	*F*	*p*	*F*	*p*
Perennial grasses	4	129.965	.000**	91.521	.000**	75.867	.000**
Extract	3	23.442	.000**	48.356	.000**	46.212	.000**
Grass × extract	12	3.256	.001**	5.920	.000**	6.263	.000**

	*df*	RL		SL		DW	
		*F*	*p*	*F*	*p*	*F*	*p*
Perennial grasses	4	877.341	.000**	2,744.101	.000**	1,208.344	.000**
Extract	3	588.545	.000**	207.173	.000**	60.348	.000**
Grass × extract	12	60.507	.000**	18.000	.000**	5.351	.000**

Abbreviations: A, species; B, Various aqueous of *Elymus nutans*; *df*, degrees of freedom.

**Significant at *p* < .01.

**FIGURE 4:**
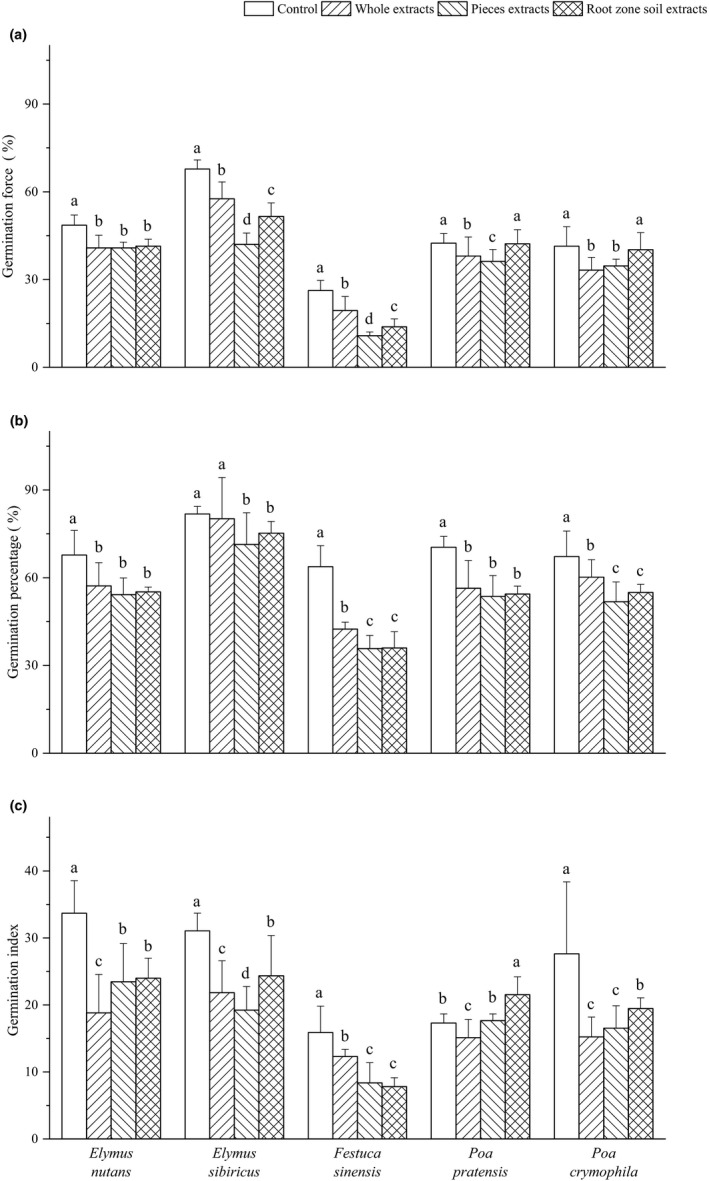
Effects of *Elymus nutans* extract on germination of highland grasses. Values are mean ± *SD* (*n* = 5). Different letters denote significant differences between treatments with a > b > c > d

The effects of the three types of extract on germination force of perennial grasses are shown in Figure 4a. The control's germination force of *Elymus sibiricus* was greater than 65% and that of other four grasses was less than 50%, suggesting *Elymus sibiricus* seed has strong vitality and the emergence is uniform. The pieces aqueous extract treatment resulted in significant decrease in germination force of all tested perennial grasses, with a biggest decrease by 58.8% of *Festuca sinensis* and smallest decrease by 14.6% of *Poa pratensis* compared with control. The pieces aqueous extract treatment leads to a significant decrease in germination force of *Elymus sibiricus*, *Festuca sinensis,* and *Poa pratensis* compared with those of whole extract treatment. Root zone soil extract had no significant allelopathic effect on germination force of *Poa pratensis* and *Poa crymophila*, but significantly lowered the germination force of *Elymus sibiricus*, *Festuca sinensis*, and *Elymus nutans* itself.

The GP of the control of the five perennial grasses was less than 70%. The GP of perennial grasses was decreased by the three kinds of extract (Figure 4b). The whole extract decreased the GP of *Elymus nutans*, *Festuca sinensis*, *Poa pratensis,* and *Poa crymophila* except for *Elymus sibiricus*. The pieces aqueous extract and root zone soil extract showed significant inhibitory on GP of *Festuca sinensis* and *Poa crymophila* compared with control and whole extract.

The germination indexes of all control were less than 40, indicating lower seed vigor of the tested perennial grasses (Figure 4c). The reduction in GI by the whole extract varied with donor plants. The biggest drop occurred in *Elymus nutans* and *Poa crymophila*. The pieces aqueous extract reduced *Festuca sinensis* (47.3%), *Poa crymophila* (40.2%), and *Elymus sibiricus* (38.1%) significantly. The root zone soil extract increased the GI of *Poa pratensis* by 24.3%, but decreased that of other four grasses.

**FIGURE 5:**
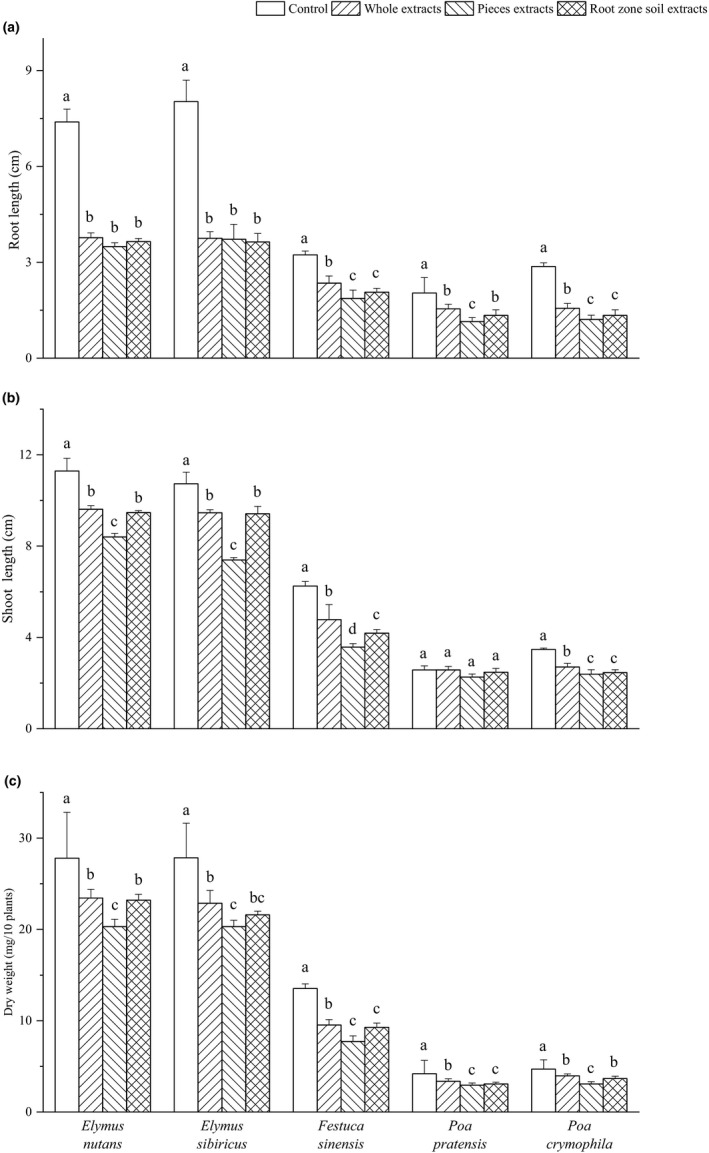
Effects of *Elymus nutans* extract on growth of highland grasses. Values are mean ± *SD* (*n* = 5). Different letters denote significant differences between treatments with a > b > c > d

The root growth of *Elymus nutans* and *Elymus sibiricus* was significantly inhibited by the three kinds of extract, and the differences among extract treatments were not significant (Figure 5a). The effects of the extract on the RL of *Festuca sinensis*, *Poa pratensis,* and *Poa crymophila* exhibited similar pattern; that is, the pieces aqueous extract and root zone soil extract presented stronger inhibition than whole extract, but a slight difference in *Poa pratensis*.

Except for *Poa pratensis*, the three kinds of extract significantly affected SL of other four donor grasses (Figure 5b). The pieces aqueous extract exhibited the strongest SL inhibition on *Festuca sinensis*, *Poa crymophila*, *Elymus sibiricus,* and *Elymus nutans*, and resulted in SL reduction by 42.9%, 31.1%, 25.6%, and 21.0%, respectively.

The overall influence of different treatments on seedling DW of donor grasses was inhibited, varied with grasses and extract (Figure 5c). The pieces aqueous extract lead to significant seedling DW decrease in *Festuca sinensis*, *Poa crymophila*, *Poa pratensis*, *Elymus sibiricus,* and *Elymus nutans* of 42.9%, 34.7%, 30.2%, 27.1%, and 26.9%, respectively. The root zone soil extract presented similar effects of the pieces aqueous extract on *Poa pratensis*, but similar effects of whole extract on other four grasses.

**FIGURE 6:**
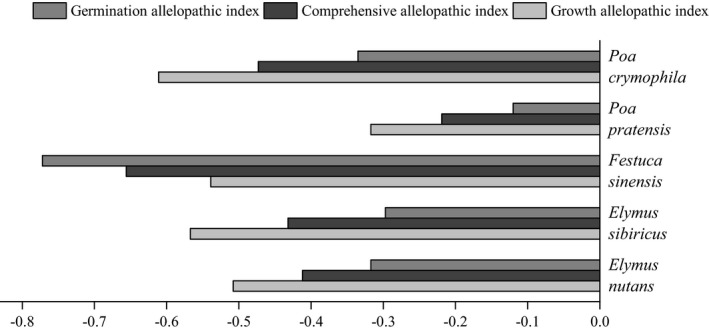
Comprehensive allelopathic effect index of *Elymus nutans* extract on tested grasses

The comprehensive allelopathic effect index of *Elymus nutans* extract on the tested grasses varied greatly (Figure 6). The maximum germination allelopathic index was noted for *Festuca sinensis* (−0.772), and the minimum was *Poa pratensis* (−0.120). The *Elymus nutans* extract mainly inhibited the germination of fescue seeds, and the root and bud growth of *Poa crymophila*, *Poa pratensis*, *Elymus sibiricus* and *Elymus nutans*.

## 4 DISCUSSIONS

Plant allelopathy is a traditional subject, and numerous studies have demonstrated that weeds can exhibit allelopathic effects on economic crops (Nichols et al., 2015). Some perennial grasses, such as *Agropyron repens*, *Lolium perenne*, *Festuca rubra,* and *Poa pratensis,* have also been proved to be allelopathic to other plants (Fales & Wakefield, 1981; Grummer, 1961). Recently, interest has developed in the allelopathy of agroecosystems, such as cultivated land and commercial forest (Kural & Zkan, 2020; Mushtaq et al., 2020). However, the role of allelopathic effects of perennial grass on the seeds and seedlings of crops and other grass species remains largely unknown. Our results indicated that the extract from *Elymus nutans* plant materials and root zone soil did impact the germination and seedling growth of commonly cultivated crops on plateau area and perennial grasses of mix‐seeded for vegetation restoration.

The effects of different extract treatments on germination varied with crops and extract types. The annual monocots were more susceptible to inhibition of the extract, while the annual dicots tended to be more tolerant or be promoted. The effects of *Elymus nutans* plant materials and root zone soil extract on the growth of seedlings were mainly manifested in inhibiting the growth of roots and shoots, and the impact on monocot crops is greater than the impact on dicot crops. Our results suggested that the three types of extract had no significant inhibition or promotion on the seedling growth of *Chenopodium quinoa*.

All tested grasses in this study were perennial, different from the aforementioned crops. The highest germination force of control was 67.8%, and the lowest was only 26.2%, reflecting the nonuniformity of germination. The germination rates of control of the five grasses ranged from 63.8% to 81.8%. Except for *Elymus sibiricus,* which was greater than 80%, the germination rate of other four species was all less than 70%. The control GI of the five grasses was all less than 35%, reflecting the lower vitality of tested perennial grass seeds.

Except that the root zone soil extract did not have a significant effect on the two *Poa* grasses, other extract treatments caused significant decrease in the germination force, germination rate, and GI of the five grasses. The strongest inhibitory effect was the pieces aqueous extract. Among the five grass species, the least affected was *Elymus sibiricus*, and the most affected was *Festuca sinensis*, which was consistent with previous reports (Liang et al., 2020). The effects of the extract on the growth of grass seedlings were mainly manifested in the inhibition of the growth of roots, especially the growth of *Elymus nutans* and *Elymus sibiricus*. The three kinds of extract had no significant effect on the seedling growth of *Poa pratensis*, but other extract had inhibitive effects on the growth of roots and shoots, as well as the DW. Particularly, the effects of the pieces aqueous extract were more apparent. From allelopathic perspective, these may be reasonable explanations for the degradation of mix‐seeded pasture in alpine area.

Previous studies on autotoxicity of forage mainly focused on alfalfa with less reports of autotoxicity about Gramineae (Ghimire et al., 2019). The present study clearly demonstrated that *Elymus nutans* had autotoxicity during their germination and seedling growth. This may help to explain rapid decline of seed yield and aboveground biomass of *Elymus nutans* monoculture after 3 years.

The differences in the same index for the same crop and forage should be attributed to different extract. The composition of the whole plant extract was analogous to that of rain leaching under natural conditions. The plant pieces aqueous extract contained more components than whole plant extract because sample ground broke plant tissues, and some enzymes, amino acids, inorganic salts, and nitrogen‐containing substances entered in the extract. The soil extract from the root zone of *Elymus nutans* contained root exudates, leachate from aboveground parts, and residues from the decomposition of dead roots in the soil, and soil microorganism‐related substances, although the amount may be a little. The composition differences of extract might contribute to the indicator differences of the same crop or forage.

## 5 CONCLUSIONS

*Elymus nutans* does have allelopathic potential on germination and seedling growth of highland crops or perennial grasses, and the overall effect is inhibited. Different crops or perennial grasses respond differently, and some are sensitive and some are tolerant. The germination force (>80%) and germination rate (>85%) of the control of five crops were relatively higher, reflecting their good germination uniformity. Of the five crops, *Hordeum vulgar* var. *nudum* and *Avena sativa* are susceptible and *Chenopodium quinoa* is tolerant. Of the five perennial grasses, *Elymus sibiricus* is the least affected and *Festuca sinensis* is the most affected. The responses of seed germination and seedling growth of the same crop or grasses vary with the extract from different sources. The pieces aqueous extract has stronger inhibition than others.

*Elymus nutans* has less allelopathic effects on *Poa pratensis* and *Poa crymophila* than on *Festuca sinensis*. It is recommended that the co‐seeding combination for restoration should consider allopathic effects of different species and reduce the seeding rate of *Elymus nutans* in mixture. The subsequent crop followed seed production of *Elymus nutans* monoculture should take planting *Chenopodium quinoa* or *Brassica napus* into consideration.

## CONFLICT OF INTEREST

None declared.

## AUTHOR CONTRIBUTIONS

**Xiaolong Quan:** Writing‐original draft (lead). **Youming Qiao:** Supervision (lead). **Mengci Chen:** Data curation (supporting). **Zhonghua Duan:** Data curation (supporting). **Huilan Shi:** Data curation (supporting).

### OPEN RESEARCH BADGES

This article has earned an Open Data and Preregistered, for making publicly available the digitally‐shareable data necessary to reproduce the reported results. The data is available at DOI https://doi.org/10.5061/dryad.tht76hdzv.

## Data Availability

Xiaolong Quan (2021), Comprehensive evaluation of the allelopathic potential of *Elymus nutans*, Dryad, Dataset, https://doi.org/10.5061/dryad.tht76hdzv
